# Maternal Pregnancy Outcomes and Offspring Risk of Adult-Onset Multiple Sclerosis

**DOI:** 10.1001/jamaneurol.2025.5255

**Published:** 2026-01-12

**Authors:** Katrin Wolfova, Bo Lars Engdahl, Julie Horn, Claire S. Riley, Natalie A. Bello, Eliza C. Miller, Sarah E. Tom

**Affiliations:** 1 Department of Neurology, Columbia University, Presbyterian Hospital, New York, New York; 2 Department of Public Health, Second Faculty of Medicine, Charles University, Prague, Czech Republic; 3 Department of Physical Health and Ageing, National Institute of Public Health, Oslo, Norway; 4 HUNT Research Centre, Department of Public Health and Nursing, Norwegian University of Science and Technology, Levanger, Norway; 5 Department of Obstetrics and Gynecology, Levanger Hospital, Nord-Trøndelag Hospital Trust, Levanger, Norway; 6 Department of Cardiology, Smidt Heart Institute, Cedars-Sinai Medical Center, Los Angeles, California; 7 Atria Health and Research Institute, New York, New York; 8 Department of Neurology, University of Pittsburgh, Pittsburgh, Pennsylvania; 9 Department of Epidemiology, Columbia University, New York, New York

## Abstract

**Importance:**

Although concordance rates for multiple sclerosis (MS) are higher among dizygotic twins than among nontwin siblings—suggesting a potential influence of prenatal and perinatal factors—few large-scale studies have systematically investigated the role of maternal adverse pregnancy outcomes in risk of MS in offspring.

**Objective:**

To explore whether preterm birth, being born small or large for gestational age (SGA and LGA, respectively), maternal hypertensive disorders of pregnancy (HDP), placental abruption, and maternal diabetes are associated with the risk of adult-onset MS in offspring.

**Design, Setting, and Participants:**

A closed cohort study with a follow-up beginning in January 2009 and continuing until the first event, death, emigration, or end of follow-up (December 2019). Data were derived from Norwegian national registers. All live births (N = 1 303 802) in the Medical Birth Registry of Norway from 1967 to 1989 were identified. MS cases were identified through the National Patient Register. Cox models were used to estimate the association between adverse pregnancy outcomes and MS among participants aged 18 years and older at the beginning of the follow-up and MS-free during the previous year. Data were analyzed from February to October 2025.

**Exposures:**

Primary exposures included preterm birth (before gestational age of 37 completed weeks), SGA (birth weight <10th percentile), LGA (birth weight >90th percentile), HDP (preeclampsia, eclampsia, gestational hypertension, and chronic hypertension), placental abruption, and maternal diabetes (type 2, unspecified pregestational diabetes, gestational diabetes, and use of antidiabetic medication during pregnancy).

**Main Outcomes and Measures:**

MS diagnosis defined by *International Classification of Diseases and Related Health Problems*, *Tenth Revision*, code G35.

**Results:**

Among 1 166 731 infants (597 330 [51.2%] male), 4295 MS cases were identified in 2009 and onwards. Adjusting for confounders, the hazard ratio [HR] for LGA was 1.13 (95% CI, 1.03-1.25), while the HR for SGA was 0.88 (95% CI, 0.78-0.98). The HR for maternal diabetes was 2.15 (95% CI, 1.37-3.37). Preterm birth, placental abruption, and HDP were not associated with MS.

**Conclusions and Relevance:**

In this cohort study, being born LGA and being exposed to maternal diabetes were associated with a higher risk of adult-onset MS, whereas begin born SGA was associated with a lower risk. While high childhood body mass index and diabetes are known MS risk factors, these findings suggest susceptibility may begin as early as the prenatal period.

## Key Points

**Question:**

Are preterm birth, being small or large for gestational age, maternal hypertensive disorders of pregnancy, placental abruption, or maternal diabetes associated with risk of adult-onset multiple sclerosis (MS) in offspring?

**Findings:**

In this cohort study among 1 303 802 participants in Norway, being born large for gestational age and exposure to maternal diabetes were associated with a higher MS risk, while being born small for gestational age was associated with a lower MS risk. Preterm birth, placental abruption, and hypertensive disorders of pregnancy were not associated with MS.

**Meaning:**

The findings suggest that MS susceptibility may begin as early as in the prenatal period.

## Teaser

This cohort study evaluates associations between various maternal pregnancy outcomes and risk of multiple sclerosis in offspring.

## Introduction

Multiple sclerosis (MS), a leading cause of neurological disability worldwide in adults younger than 50 years, stems from chronic immune dysregulation caused by complex interactions between genetic and environmental factors.^1^ The concordance rates for monozygotic twins (25%-30%) support a genetic contribution to MS susceptibility, while the higher concordance rates for dizygotic twins (5%-14%) compared to nontwin siblings (3%) suggest a role of prenatal and perinatal exposures.^2,3^ Adverse pregnancy outcomes (APOs)—encompassing preterm birth, restrictive or excessive fetal growth, hypertensive disorders of pregnancy (HDPs), placental abruption, and gestational diabetes—have been associated with altered immune development and may influence long-term MS risk in the offspring.^4,5,6^

In Northern Europe, where MS prevalence is among the highest globally, these key APOs occur with moderate frequency. For example, in Norway, around 6.2% infants are born preterm, HDPs are diagnosed in 5.8% of pregnancies and preeclampsia in 2.7% to 4.3%, and the prevalence of gestational diabetes ranges between 2.0% and 4.6% in Norway compared to 5.2% to 13.7% in the US and Canada.^7,8,9,10,11^ Fetal growth abnormalities, defined using small or large for gestational age (SGA and LGA, respectively) thresholds, by definition affect approximately 10% of births at each end of the growth spectrum, but Norwegian infants tend to be larger than those in Southern European populations.^12^

Epidemiological evidence demonstrates that maternal APOs are associated with offspring cardio-kidney-metabolic syndrome later in life.^13,14,15^ While cardiovascular and metabolic conditions and MS may share common pathways—for example, via the immune system dysregulation—whether maternal APOs influence the risk of MS in the offspring remains insufficiently explored. Some studies found an increased risk of MS in offspring of mothers with diabetes and with low gestational vitamin D levels.^16,17^ A previous study of US nurses reported null associations between MS risk and being born 2 weeks premature, both low and high birth weight, and preeclampsia,^18^ and higher MS risk among offspring of mothers with diabetes.^18^ However, the study relied on retrospectively collected, maternally reported data, was likely underpowered to detect associations for several exposures, and did not examine fetal growth for gestational age. We conducted a population-based study of more than 1 million births in Norway to explore whether maternal APOs were associated with a risk of adult-onset MS in offspring.

## Methods

### Ethics

The study was approved by the Regional Committees for Medical and Health Research Ethics. The dataset received by the researchers was pseudonymized. Register-based studies using pseudonymized data are exempt from consent requirements according to Norwegian data protection legislation.

### Study Design

This closed cohort study uses Norwegian nationwide registry data. We followed the Strengthening the Reporting of Observational Studies in Epidemiology (STROBE) reporting guideline.

### Data Sources and Linkage

We linked data from the Norwegian Central Population registry, the National Educational Data Base,^19^ and 3 compulsory Norwegian national health registers: the Norwegian Cause of Death Registry (dating back to 1951), the Medical Birth Registry of Norway (1967), and the Norwegian Patient Registry (2008). All nationals and residents of Norway are assigned a unique 11-digit personal identification number. All registries were updated through December 2019 and the unique personal identification was used to link data between registers. The Medical Birth Registry of Norway contains prospectively collected data on pregnancy, delivery, and maternal and neonatal health reported by midwifes or by the mother’s physician.^20^ The Norwegian Patient Registry contains diagnoses (*International Classification of Diseases*, *10th Revision* [*ICD-10*]), reported from both public and private inpatient and outpatient specialist health care providers.^21^ The Norwegian Cause of Death registry includes data for time of death.^22^ We used parental information from the Norwegian Central Population Registry to identify siblings and to obtain data on parental education by linking the data to the National Education Database.

### Sample

We identified all live births in Norway from 1967 to 1989 (N = 1 303 802) (eFigure 1 in Supplement 1). Of those, 68 492 people (5.0%) were not included in the analytic sample as 37 483 were no longer living in Norway in 2008, and 31 009 had died prior to that year. Individuals with missing data on gestational age (5.3% of births) and birth weight (0.1%) were also excluded. We further excluded those with birth weight *z* score less than 4.0 or greater than 4.0 (n = 672) to avoid misclassification^23^ and those with type 1 maternal diabetes (n = 32), as genetic factors can partially explain the co-occurrence of type 1 diabetes and MS.^24^ For the analysis of incident MS cases, the follow-up began in January 2009, when participants were at least 18 years old and MS-free during the previous year, and continued until the first event, death, emigration, or end of follow-up (December 2019). The analytic sample for the analysis of incident MS (ie, excluding MS cases diagnosed in 2008) consisted of 1 166 731 individuals. To evaluate robustness of our findings, we additionally included 1483 participants who had a record of MS diagnosis in 2008, which might include cases diagnosed at any point during the life course. The sample for the analysis of prevalent MS between 2008 and 2019 comprised 1 168 214 individuals. For an analysis of siblings, we identified 9889 individuals from 3984 families in which at least 1 sibling was diagnosed with MS while another remained MS-free.

### Exposures

Information on gestational age, indicated by the last menstrual period, birth weight, HDPs, placental abruption, and maternal diabetes was retrieved from the Medical Birth Registry of Norway. Preterm birth (yes vs no) was defined as medically indicated or spontaneous birth before the gestational age of 37 completed weeks. We calculated birth weight for gestational age, indicative of restrictive or excessive fetal growth, based on the sample distribution, separately for each sex. SGA was defined as birth weight less than the 10th percentile, LGA as birth weight greater than the 90th percentile, and 10th to 90th percentile as appropriate-for-gestational-age (AGA). HDP (yes vs no) included preeclampsia, eclampsia, gestational hypertension, and chronic hypertension. Maternal diabetes (yes vs no) included type 2 diabetes, unspecified pregestational diabetes, gestational diabetes, and use of any antidiabetic medication during pregnancy only. We created alternative definitions of the exposures as follows: we (1) calculated birth weight for gestational age using Norwegian sex-specific reference curves (1967-1998) and created a categorical variable with 3 levels: SGA (birth weight <10th percentile), LGA (birth weight >90th percentile), and AGA (10th to 90th percentile)^25^; (2) included only de novo HDPs and excluded individuals with maternal chronic hypertension; and (3) included only maternal gestational diabetes and excluded individuals with other types of maternal diabetes from the sample.

### Outcome

The outcome was MS diagnosis defined by *ICD-10* code G35 in the Norwegian Patient Registry from 2008 to 2019. The positive predictive value for MS diagnosis in the Norwegian Patient Registry is 92% and the negative predictive value is 99%.^26^ The year of the first diagnosis event was registered. We defined an alternative outcome as requiring at least two MS *ICD* code occurrences during the study period.

### Covariates

Year of birth, birth cohort (1967-1977 vs 1978-1989), sex, and maternal country of origin (Norway, other high-income countries, or middle- and low-income countries) were obtained from the Norwegian Central Population registry. Mother’s age at the time of delivery, number of previous children (1, 2, 3, 4, or ≥5) and birth plurality (singleton vs multiple) were obtained from the Medical Birth Registry of Norway. Maternal education (primary, secondary, or tertiary) was obtained from the National Education Data Base. Latitude was defined as being born in 1 of the 5 counties with the highest latitude vs all other counties. All variables are listed in eTable 1 in Supplement 1.

### Statistical Analysis

We used Cox proportional hazards regression models to estimate hazard ratios (HRs) and their corresponding 95% CIs for the association between each exposure and incident MS, excluding cases diagnosed in 2008. Age was used as the time axis. Individuals were followed from 2009 until the year of diagnosis, death, emigration, or the end of follow-up, whichever occurred first. Cox models were adjusted for sex, birth cohort, mother’s age at the time of delivery, previous children, birth plurality, maternal education, and maternal country of origin. Models with preterm birth and birth weight for gestational age as the exposures were additionally adjusted for HDPs, placental abruption, and maternal diabetes. Models with placental abruption as the exposure were additionally adjusted for HDP and maternal diabetes. Proportional hazards assumptions were met in all Cox models (global Schoenfeld test, *P* > .05).

To correct for selection bias due to conditioning on surviving and not emigrating until 2008, we weighted the survival analyses by the inverse probability of surviving and not emigrating. We calculated the inverse probability weights by predicting being alive and not emigrated by January 1, 2008, using a logistic regression model that included the following variables that may affect survival but not necessarily the outcome: sex, maternal education, calendar year, gestational age, birth weight, HDPs, placental abruption, maternal diabetes, birth defects, maternal age, number of previous children, and birth plurality. To estimate the cumulative incidence function of MS while accounting for death as a competing event during follow-up, we fitted Fine and Gray models.^27^

To explore associations between each exposure and MS in a broader set of cases, we additionally applied logistic regression models to estimate odds ratios (ORs) and 95% CIs in the prevalence sample, adjusting for the same set of confounders, with calendar year of birth as a continuous variable. Both ORs and HRr approximate risk ratio when the outcome is rare (<10%).^28^

To take into account unmeasured confounding factors shared by siblings (eg, genetic factors and parental characteristics), we applied a sibling-comparison design in the prevalence sample. To estimate the association with MS, we fitted a conditional logistic regression, also referred to as matched case-control model, restricted to only families with 1 or more siblings with an MS diagnosis (ie, cases) and 2 or more siblings without an MS diagnosis (ie, controls).

In addition, we studied the linear association between birth weight and MS and between gestational age and MS separately, and we tested for possible nonlinear associations by fitting restricted cubic splines. We checked the robustness to alternative definitions of the outcome and the exposures as defined above. To check the robustness to additional potential confounding, we further adjusted for latitude.

To evaluate the extent of potential unmeasured confounding, we calculated the E-value for each exposure, assuming rare outcomes. The E-value indicates how strongly an unmeasured confounder, or a set of confounders, would need to be associated with both the exposure and the outcome to explain away the observed association.^29^ Analyses were performed using Stata version 16.0 (StataCorp). Two-tailed *P* values less than .05 were considered statistically significant. Data were analyzed from February to October 2025.

## Results

Among 1 166 731 infants, 597 330 (51.2%) were male and 569 401 (48.8%) were female. A total of 57 987 (5.0%) participants were born preterm; 114 865 (9.8%) participants were born SGA; 114 025 (9.8%) participants were born LGA; and 49 598 (4.2) participants were exposed to maternal HDPs, 4778 (0.4%) to maternal placental abruption, and 2662 (0.2%) to maternal diabetes (eTable 2 in Supplement 1). During the 14 880 998 person-years of follow-up since 2009, 4295 MS cases were identified between 2009 and 2019, and additional 1483 MS cases were identified in 2008. The median (IQR) age at diagnosis was 37 (32-42) years. A total of 570 458 (48.8%) participants were female, and a total of 4036 (69.8%) participants were female among MS cases. Participant characteristics according to each exposure are presented in eTables 2 and 3 in Supplement 1.

Adjusting for confounders, the HR for being LGA vs AGA was 1.13 (95% CI, 1.03-1.25), while for being SGA it was 0.88 (95% CI, 0.78-0.98) in the incidence sample (Table 1). The results remained similar after additional adjustment for HDPs, placental abruption, and maternal diabetes (Table 1). Accounting for death prior to the study period and after the start of follow-up, the HRs in individuals who were SGA and LGA were nearly identical as the HRs in the main analysis (Figure; eFigure 2 in Supplement 1). The association between being LGA and MS was attenuated in the prevalence sample (OR, 1.09; 95% CI, 1.00-1.19), while the association between being SGA and MS in the prevalence sample remained similar (OR, 0.87; 95% CI, 0.79-0.96), and adjusting for additional confounders did not alter the results (Table 1). The point estimates in the sibling analysis were similar, however, the confidence intervals showed greater uncertainty (Table 1). Using the definition of birth weight for gestational age based on national charts, the results remained essentially unchanged (Table 2).

**Table 1.** Associations Between Maternal Adverse Pregnancy Outcomes and the Risk of Multiple Sclerosis in the Offspring

Exposure	Population analysis						Sibling analysis, prevalence sample (n = 9889)		
	Incidence sample (n = 1 166 731)			Prevalence sample (n = 1 168 214)					
	Events, No.	Adjusted HR (95% CI)^a^	Additionally adjusted HR^a^^,^^b^	Events, No.	Adjusted OR (95% CI)^c^	Additionally adjusted OR^b^^,^^c^	Events, No.	Adjusted OR (95% CI)^c^	Additionally adjusted OR^b^
Preterm birth									
Yes	198	0.99 (0.85-1.14)	0.97 (0.83-1.12)	254	0.92 (0.81-1.05)	0.91 (0.80-1.04)	169	1.04 (0.82-1.32)	1.05 (0.83-1.34)
No	4114	1 [Reference]	1 [Reference]	5546	1 [Reference]	1 [Reference]	3914	1 [Reference]	1 [Reference]
Birth weight for gestational age									
SGA	369	0.88 (0.78-0.98)	0.88 (0.79-0.98)	502	0.87 (0.79-0.96)	0.88 (0.80-0.96)	343	0.89 (0.74-1.07)	0.90 (0.75-1.08)
AGA	3469	1 [Reference]	1 [Reference]	4679	1 [Reference]	1 [Reference]	3285	1 [Reference]	1 [Reference]
LGA	474	1.13 (1.03-1.25)	1.13 (1.02-1.24)	619	1.09 (1.00-1.19)	1.09 (1.00-1.19)	455	1.11 (0.94-1.30)	1.10 (0.94-1.30)
Placental abruption									
Yes	21	1.17 (0.75-1.84)	1.167(0.75-1.82)	23	0.98 (0.64-1.51)	0.99 (0.64-1.52)	10	0.63 (0.28-1.39)	0.63 (0.29-1.40)
No	4291	1 [Reference]	1 [Reference]	5777	1 [Reference]	1 [Reference]	4073	1 [Reference]	1 [Reference]
HDP									
Yes	160	0.90 (0.76-1.05)	NA	215	0.92 (0.80-1.05)	NA	147	0.76 (0.59-0.99)	NA
No	4152	1 [Reference]	NA	5585	1 [Reference]	NA	3936	1 [Reference]	NA
Maternal diabetes									
Yes	19	2.15 (1.37-3.37)	NA	23	1.99 (1.32-3.01)	NA	11	1.17 (0.33-4.19)	NA
No	4293	1 [Reference]	NA	5777	1 [Reference]	NA	4072	1 [Reference]	NA

Abbreviations: AGA, appropriate for gestational age; HDPs, hypertensive disorders of pregnancy; HR, hazard ratio; LGA, large for gestational age; NA, not applicable; OR, odds ratio; SGA, small for gestational age.

^a^ Cox proportional hazard models were adjusted for sex, birth cohort (1967-1977 vs 1978-1989), mother’s age at the time of delivery, previous children, birth plurality, maternal education, and maternal country of origin.

^b^ Models with preterm birth and birth weight for gestational age as the exposures were additionally adjusted for hypertensive disorders of pregnancy, placental abruption, and maternal diabetes. Models with placental abruption as the exposure were additionally adjusted for hypertensive disorders of pregnancy and maternal diabetes.

^c^ Logistic regression models were adjusted for calendar year of birth as a continuous variable, sex, mother’s age at the time of delivery as a continuous variable, previous children (1, 2, 3, 4, ≥5), birth plurality (singleton vs multiple), maternal education (primary, secondary, and tertiary), and maternal country of origin (Norway, other high-income country, and middle- or low-income country).

**Figure.**
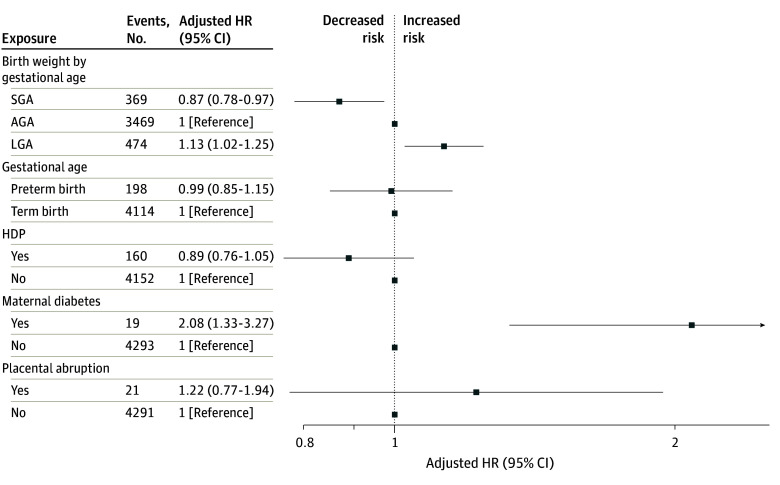
Associations Between Maternal Adverse Pregnancy Outcomes and the Risk of Multiple Sclerosis in the Offspring Accounting for Mortality (N = 1 166 731) Models were adjusted for sex, birth cohort (1967-1977 vs 1978-1989), mother’s age at the time of delivery, previous children, birth plurality, maternal education, and maternal country of origin. AGA indicates appropriate for gestational age; HDP, hypertensive disorders of pregnancy; HR, hazard ratio; IPW, inverse-probability weighted; LGA, large for gestational age; SGA, small for gestational age.

**Table 2.** Associations Between Maternal Adverse Pregnancy Outcomes and the Risk of Multiple Sclerosis in the Offspring Using Alternative Exposure Definitions

Exposure	Incidence sample (n = 1 166 731)		Prevalence sample (n = 1 168 214)	
	Events, No.	Adjusted HR (95% CI)^a^	Events, No.	Adjusted OR (95% CI)^b^
Birth weight for gestational age^c^				
SGA	441	0.87 (0.79-0.97)	598	0.88 (0.80-0.96)
AGA	3598	1 [Reference]	4845	1 [Reference]
LGA	273	1.13 (1.00-1.28)	357	1.09 (0.98-1.22)
De novo HDP^d^				
Yes	157	0.91 (0.77-1.07)	211	0.92 (0.80-1.06)
No	4155	1 [Reference]	5589	1 [Reference]
Maternal gestational diabetes^e^				
Yes	4	1.97 (0.74-5.25)	5	1.86 (0.77-4.50)
No	4293	1 [Reference]	5777	1 [Reference]

Abbreviations: AGA, appropriate for gestational age; HDP, hypertensive disorders of pregnancy; HR, hazard ratio; LGA, large for gestational age; OR, odds ratio; SGA, small for gestational age.

^a^ Cox proportional hazard models were adjusted for sex, birth cohort (1967-1977 vs 1978-1989), mother’s age at the time of delivery, previous children, birth plurality, maternal education, and maternal country of origin.

^b^ Logistic regression models were adjusted for calendar year of birth as a continuous variable, sex, mother’s age at the time of delivery as a continuous variable, previous children (1, 2, 3, 4, ≥5), birth plurality (singleton vs multiple), maternal education (primary, secondary, and tertiary), and maternal country of origin (Norway, other high-income country, and middle- or low-income country).

^c^ Birth weight for gestational age was calculated using Scandinavian sex-specific reference curves.

^d^ Individuals with maternal chronic hypertension were excluded from the sample.

^e^ Individuals with other types of maternal diabetes were excluded from the sample.

Adjusting for confounders, the HR for maternal diabetes was 2.15 (95% CI, 1.37-3.37) in the incidence sample and the odds of MS were higher (OR 1.99; 95% CI, 1.32-3.01) in the prevalence sample (Table 1). Accounting for death prior to the study period and during the study period did not alter the results (Figure). We did not find an association between maternal diabetes and MS in the sibling analysis, but the estimates were imprecise due to a small number of MS cases in this group (OR, 1.17; 95% CI 0.33-4.19) (Table 1). In the model with maternal gestational diabetes as the exposure, the point estimate was higher (OR, 1.86; 95% CI 0.77-4.50) (Table 2), but the uncertainty around the estimate was still large, as there were only 5 MS cases in the exposed group. Preterm birth, placental abruption, and HDPs were not associated with MS (Table 1).

Using the alternative outcome defined as at least 2 MS-related *ICD* codes occurrence during the study period changed the associations by 4% or less (eTable 4 in Supplement 1). We found a linear association between birth weight and the incidence (HR, 1.07; 95% CI, 1.04-1.11) and prevalence of MS (OR, 1.08; 95% CI, 1.05-1.11 per standard deviation of birth weight). Gestational age alone was not associated with either the incidence of (HR, 1.00; 95% CI, 0.98-1.01) or the prevalence (OR, 1.00; 95% CI, 0.99-1.01). Adjusting for diabetes did not alter the associations. Fitting birth weight (eFigure 3 in Supplement 1) and gestational age with cubic splines with 4 knots did not result in a better model fit (log likelihood *P* > .05). Although high latitude was associated with MS (OR, 1.16; 95% CI, 1.10-1.23), adding latitude as a confounder changed the effect estimates by 2% or less. E-values ranged from 1.40 to 3.72 across exposures, indicating mild to moderate robustness to unmeasured confounding (eTable 5 in Supplement 1).

## Discussion

In this cohort study of national registry data, exposure to maternal diabetes and birth as LGA were associated with a higher risk of adult-onset MS, whereas being born SGA was associated with a lower risk compared to AGA. Consistent with previous reports, we did not find a higher MS risk among those who were born preterm and those who were exposed to HDPs or placental abruption. Mortality before or during the study period did not influence these associations.

Exposure to maternal diabetes during prenatal period was first suggested as an MS risk factor by Gardener at al.^18^ They found a 10-fold higher MS rate among offspring of mothers with diabetes, but the study included only 2 exposed MS cases based on self-reported exposure.^18^ We found a 2-fold higher rate of MS among offspring of mothers with any type of diabetes, excluding type 1 due to its potential genetic association with MS. However, the association between maternal gestational diabetes and offspring MS risk was not statistically significant, potentially due to a small sample of only 5 MS cases in this group. A nationwide study^16^ of all singletons born in Denmark from 1978 to 2008 supports our findings. While the study found a similar MS rate among individuals born to mothers without diabetes and those exposed to gestational diabetes, individuals whose mothers had pregestational diabetes had a 2-fold higher rate after controlling for maternal obesity, and the rate was slightly greater among those exposed to maternal pregestational type 2 diabetes compared to type 1.^16^ We did not have data on maternal obesity. However, the chance of residual confounding is small, as the unmeasured confounder would have to confer a 3.72 higher risk of both maternal diabetes and MS to explain away the observed association.

The underlying mechanism remains unclear. One possibility is that exposure to maternal hyperglycemia leads to lasting immunological changes by altering immune system programming.^30^ Alternatively, maternal diabetes may increase the likelihood of higher birth weight and elevated childhood body mass index (BMI) in offspring,^31^ which then increases the risk of MS possibly through chronic low-grade inflammation or lower circulating levels of vitamin D metabolites.^32^

Our findings do not support previous reports of null effects of either birth weight, or birth weight for gestational age, on the risk of MS.^18,33,34^ Counterintuitively, we found that being born SGA was associated with a lower risk of developing MS later in life, despite SGA being a known risk factor for metabolic and cardiovascular diseases in adulthood,^35^ which are themselves linked to MS risk and progression.^36,37,38^ The higher MS risk among those born LGA is more expected and in line with a case-control study from Argentina where female individuals with MS had 4.5-times higher odds of birth weight of more than 4000 g and male individuals with MS had 6-times higher odds.^39^ Individuals who are SGA and LGA may experience differences in BMI trajectories during childhood, which may then influence the MS risk. While LGA is associated with early weight gain and childhood obesity,^40^ which is a well-established risk factor for MS,^41^ evidence on SGA is less consistent, with several studies reporting lower childhood BMI in this group,^42,43,44^ which could contribute to lower MS risk. Alternatively, there may be a critical window in early life during which adiposity has a stronger influence on MS risk—effects that later changes in BMI may not reverse. Supporting this, a large cohort study from Sweden^45^ found that while individuals with pediatric obesity had double the risk of developing MS by age 30 years, lowering BMI during at least 1 year of obesity treatment did not reduce that risk. Future work should examine to what extent childhood adiposity may mediate the observed associations.

Gestational age at birth, as well as exposure to maternal HDPs and placental abruption, were not associated with an increased risk of developing MS. These findings are consistent with those from a previous case-control study in Canada^46^ and a prospective cohort study of US nurses.^18^

### Strengths and Limitations

A strength of our study is inclusion of more than 1 million individuals, a long follow-up period, and a population-based nature. As we used registry data, we minimized loss to follow-up. A potential limitation of this study is the possible misclassification of APOs, as the number of reported exposures, particularly maternal diabetes, was lower than previously documented prevalence in similar cohorts.^7,8,9,10,11^ A higher occurrence of APOs observed among later-born participants may suggest improved diagnostic procedures and, thus, more accurate APO classification, but it may also reflect a true increase in the prevalence of APOs. We cannot rule out residual confounding, as the E-values were relatively small, except for maternal diabetes, and were not able to control for maternal obesity, smoking, or vitamin D status. However, adding an indicator of high latitude to our models did not change results. Further, given the modest and partly separate maternal and fetal genetic contributions to APOs,^47,48,49^ along with the distinct genetic pathways involved in APOs and MS,^50,51^ substantial genetic confounding seems unlikely. Changes in MS diagnostic practice may have introduced nondifferential misclassification, which would likely bias the results toward the null. Although gestational age in our sample was consistently based on last menstrual period and not on ultrasonography, limiting concerns about differential misclassification, evolving clinical practices, such as use of antenatal corticosteroids and surfactant treatment, and increase in maternal obesity may have led to temporal changes in the occurrence of APOs. The generalizability of our findings may be limited. Fetal growth patterns in Northern Europe diverge from international standards: while 10.4% of live births are classified as SGA using national charts in Norway, only 3.9% meet the SGA criteria when international charts are applied.^12^ Conversely, 23.3% of infants are classified as LGA using international charts, compared to 11.1% based on national standards.^12^

## Conclusions

While it is well established that children with high BMI and diabetes are more likely to develop MS in adulthood, our findings suggest that the roots may lie in the perinatal period. Early metabolic exposures may influence immune system programming and future growth trajectories. Future epidemiologic studies should examine markers of neonatal adiposity and growth to better understand how early-life factors shape MS risk.

## Notes

Supplement 1. eMethods. Additional descriptive statistics eFigure 1. Flow chart eFigure 2. Log-odds of multiple sclerosis with birthweight fitted with cubic splines eFigure 3. Associations between maternal adverse pregnancy outcomes and the risk of multiple sclerosis in the offspring accounting for the competing risk of death during follow-up (N=1 166 731) eTable 1. List of variables eTable 2. Participants’ characteristics according to hypertensive disorders of pregnancy, placental abruption and maternal diabetes eTable 3. Participants’ characteristics according to hypertensive disorders of pregnancy, placental abruption and maternal diabetes eTable 4. Associations between maternal adverse pregnancy outcomes and the risk of multiple sclerosis in the offspring using alternative case definition (least 2 ICD code occurrences during the study period) eTable 5. E-values eReferences

Supplement 2. Data sharing statement
