# Supplementary material for: Maternal Pregnancy Outcomes and Offspring Risk of Adult-Onset Multiple Sclerosis
Source: JAMA Neurol. 2026 Jan 12;83(2):153–60. doi: 10.1001/jamaneurol.2025.5255 (PMC12797129; doi:10.1001/jamaneurol.2025.5255)
Supplement: Supplement 1. — eMethods. Additional descriptive statistics eFigure 1. Flow chart eFigure 2. Log-odds of multiple sclerosis with birthweight fitted with cubic splines eFigure 3. Associations between maternal adverse pregnancy outcomes and the risk of multiple sclerosis in the offspring accounting for the competing risk of death during follow-up (N=1 166 731) eTable 1. List of variables eTable 2. Participants’ characteristics according to hypertensive disorders of pregnancy, placental abruption and maternal diabetes eTable 3. Participants’ characteristics according to hypertensive disorders of pregnancy, placental abruption and maternal diabetes eTable 4. Associations between maternal adverse pregnancy outcomes and the risk of multiple sclerosis in the offspring using alternative case definition (least 2 ICD code occurrences during the study period) eTable 5. E-values eReferences [file jamaneurol-e255255-s001.pdf]

Supplemental Online Content

Wolfova K, Engdahl BL, Horn J, et al. Maternal pregnancy outcomes and offspring risk of adult-onset multiple sclerosis. *JAMA Neurol*. Published online January 12, 2026. doi:10.1001/jamaneurol.2025.5255

eMethods. Additional descriptive statistics
eFigure 1. Flow chart
eFigure 2. Log-odds of multiple sclerosis with birthweight fitted with cubic splines
eFigure 3. Associations between maternal adverse pregnancy outcomes and the risk of multiple sclerosis in the offspring accounting for the competing risk of death during follow-up (N=1 166 731)
eTable 1. List of variables
eTable 2. Participants' characteristics according to hypertensive disorders of pregnancy, placental abruption and maternal diabetes
eTable 3. Participants' characteristics according to hypertensive disorders of pregnancy, placental abruption and maternal diabetes
eTable 4. Associations between maternal adverse pregnancy outcomes and the risk of multiple sclerosis in the offspring using alternative case definition (least 2 ICD code occurrences during the study period)
eTable 5. E-values
eReferences

This supplemental material has been provided by the authors to give readers additional information about their work.

© 2026 Wolfova et al. *JAMA Neurology*.

Additional descriptive statistics

Group differences
Multiple births were more common across all adverse pregnancy outcomes (APO), particularly among those born preterm compared to those born at term (12.3% vs. 1.4%). Those who were born between 1978 and 1989 were more likely to be exposed to hypertensive disorders of pregnancy (HDP, 55.4% vs 44.6%), placental abruption (58.2% vs 41.8%) and maternal diabetes (67.7% vs 32.3%) compared to those born between 1967 and 1977. The proportion of females was slightly higher across all exposures. A higher proportion of mothers who experienced preterm birth, small for gestational age (SGA), large for gestational age (LGA), and placental abruption had only primary education compared to those who did not experience these events, whereas maternal educational attainment did not differ between those with and without HDP and mothers with diabetes during pregnancy had slightly higher levels of education.

Mortality
The number of deaths during the study period was 10 329 (0.8%), and the proportion of individuals with APO who died during the study period was similar to number of deaths in the whole sample (eTable 4). However, individuals who were born preterm and those whose mother's experienced placental abruption or diabetes were more likely to die before the study period (eTable 4). Maternal APO were not more frequent among those who emigrated before the study start nor during the study period (eTable 4), but birth defects were slightly more common among individuals with maternal APO compared to the general population (eTable 4).

© 2026 Wolfova et al. *JAMA Neurology*.

Figures

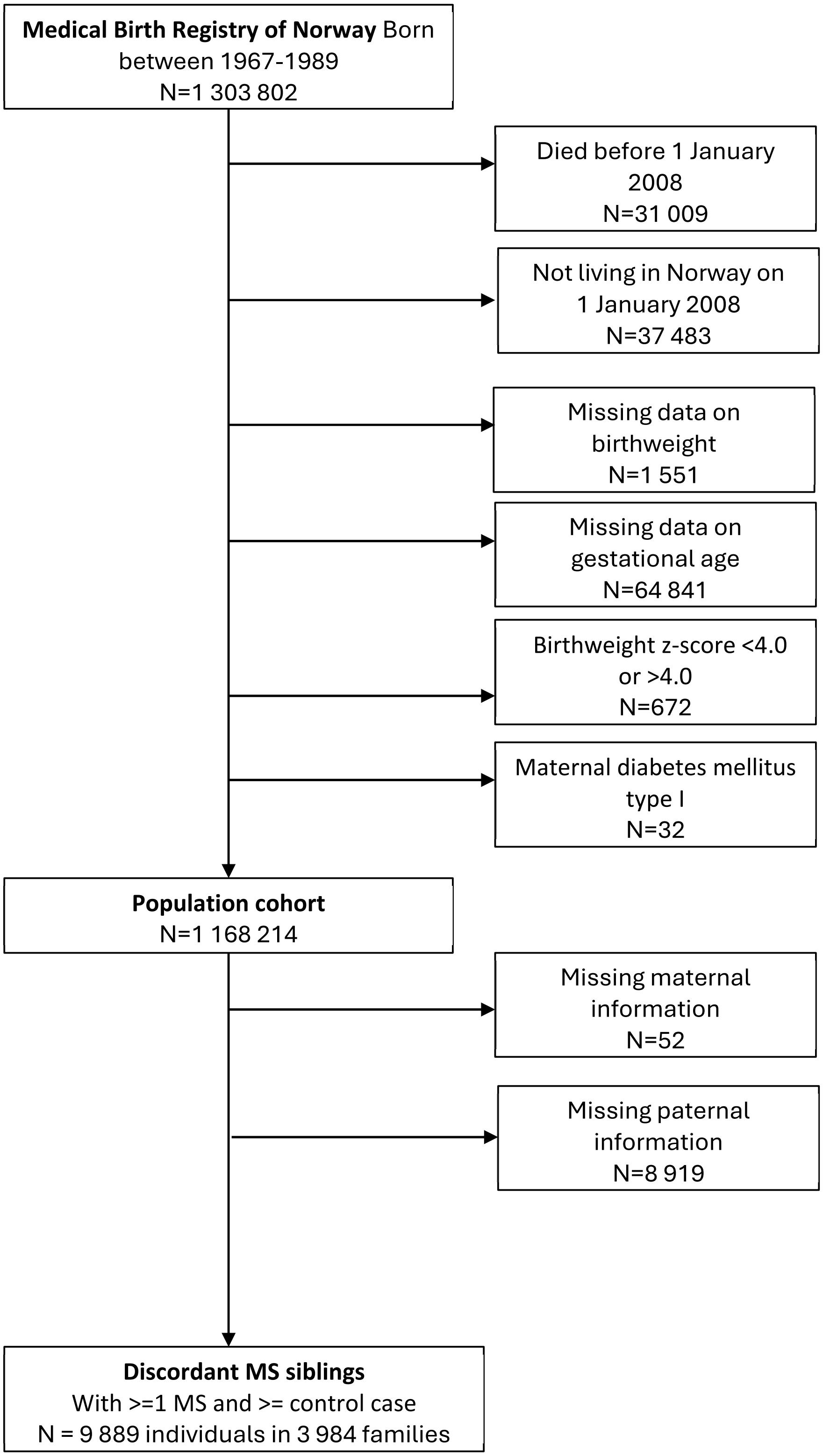

eFigure 1. Flow chart

© 2026 Wolfova et al. *JAMA Neurology*.

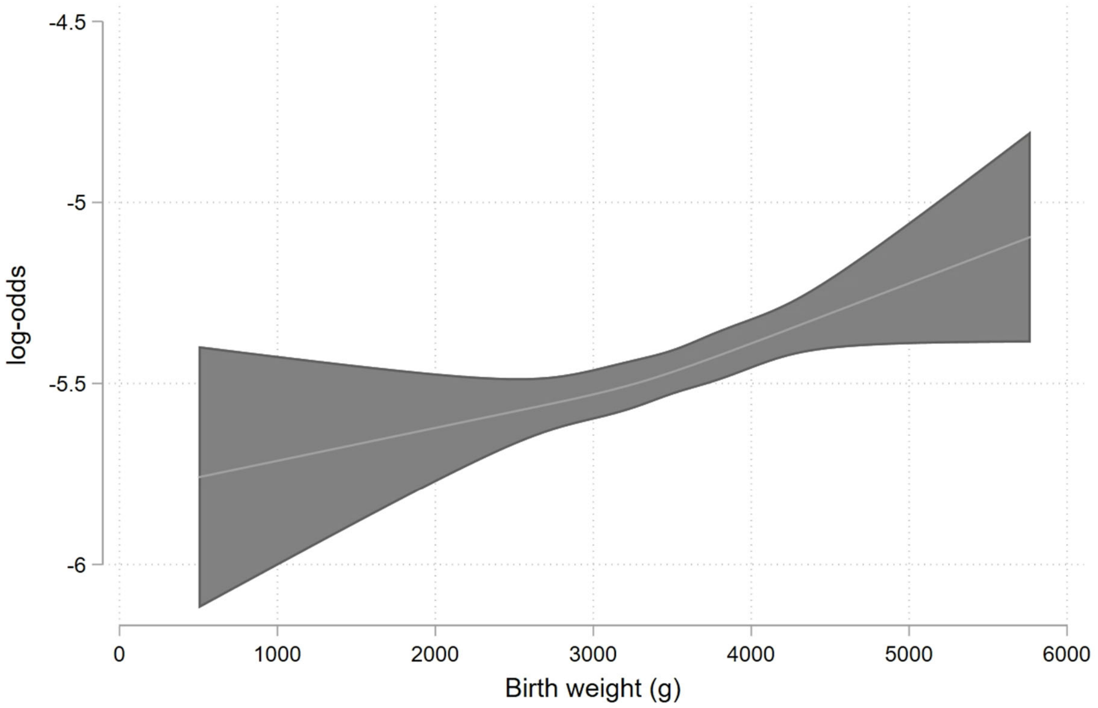

eFigure 2. Log-odds of multiple sclerosis with birthweight fitted with cubic splines

Logistic regression adjusted for calendar year of birth as a continuous variable, sex (female vs. male), mother's age at the time of delivery as a continuous variable, previous children (1, 2, 3, 4, 5+), birth plurality (singleton vs. multiple pregnancy), maternal education (primary, secondary and tertiary), and maternal country of origin (Norway, other high income country, middle and low income country).

© 2026 Wolfova et al. *JAMA Neurology*.

Exposure	Events, No.	Adjusted sHR (95% CI)
**Birth weight by gestational age**		
SGA	369	0.87 (0.77–0.97)
AGA	3469	1 [Reference]
LGA	474	1.13 (1.03–1.25)
**Gestational age**		
Preterm birth	198	0.99 (0.85–1.14)
Term birth	4114	1 [Reference]
**HDP**		
Yes	160	0.90 (0.76–1.05)
No	4152	1 [Reference]
**Maternal diabetes**		
Yes	19	2.14 (1.36–3.36)
No	4293	1 [Reference]
**Placental abruption**		
Yes	21	1.17 (0.75–1.84)
No	4291	1 [Reference]

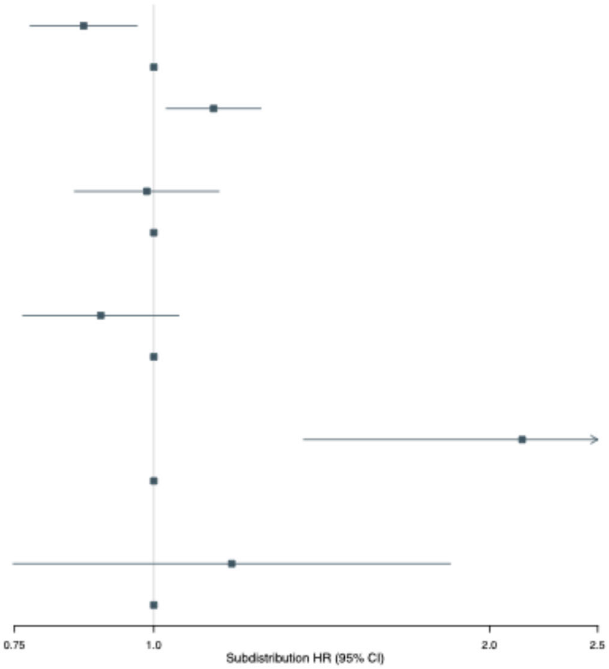

eFigure 3. Associations between maternal adverse pregnancy outcomes and the risk of multiple sclerosis in the offspring accounting for the competing risk of death during follow-up (N=1 166 731)

Abbreviations. HR = hazard ratio, CI = confidence interval, SGA = small for gestational age, AGA = appropriate for gestational age, LGA = large for gestational age, HDP = hypertensive disorders of pregnancy

Models were adjusted for sex, birth cohort (1967-1977 vs. 1978-1989), mother's age at the time of delivery, previous children, birth plurality, maternal education, and maternal country of origin.

© 2026 Wolfova et al. *JAMA Neurology*.

Tables

eTable 1. List of variables

Exposures		Registry	Variable	Notes
Preterm birth		Medical Birth Registry of Norway (MBRN)	SVLEN	Defined as medically indicated or spontaneous birth before 37 weeks of gestation. The gestational age was based on the reported date of the last menstrual period. Gestational age was missing in 5.3% cases.
Hypertensive disorder of pregnancy	Gestational hypertension	MBRN	HYPERTENSJON_ALENE	
	Preeclampsia	MBRN	PREEKL,<br>PREEKLTIDL	Until 1998, preeclampsia was recorded in MBRN either as “preeclampsia” or the combination of “hypertension” and “proteinuria”.[1]
	Chronic hypertension prior to pregnancy	MBRN	HYPERTENSJON_KRONISK	
Placental abruption		MBRN	ABRUPTIOP	
Fetal growth (SGA/AGA/LGA)		MBRN<br>Norwegian Central Population registry	VEKT<br>SVLEN<br>Sex	Birthweight for gestational age, separately for each sex. Gestational age was based on the reported date of the last menstrual period. Gestational age was missing in 5.3% and birthweight was missing in 0.1% of the births of the final sample.
Maternal gestational diabetes		MBRN	DIABETES_MELLITUS	Level 4 in variable DIABETES_MELLITUS.[2]
Maternal diabetes		MBRN	DIABETES_MELLITUS	All types of diabetes except for type 1 (level 2 – Type 2, level 3 – pregestational diabetes – unspecified, level 4 – gestational diabetes, level 5 – antidiabetic medication).

© 2026 Wolfova et al. *JAMA Neurology*.

**Outcome**			
Multiple sclerosis	Norwegian Patient Registry		ICD-10 code G35
**Covariates**			
Year of birth	Norwegian Central Population registry		
Sex	Norwegian Central Population registry		
Mother's age at the time of delivery	MBRN		
Number of previous children	MBRN		
Birth plurality	MBRN		
Maternal education	Norwegian Central Population registry and National Education Data Base		Mothers were identified through the Norwegian Central Population Registry linked to the National Education Data Base. Highest obtained education at the time of delivery was the nearest level of education in data from censuses in 1960, 1970, 1980 and yearly from 1980-1989. The variable was imputed in years in between.
Maternal country of origin	Norwegian Central Population registry		
Latitude	Norwegian Central Population registry		
**Other**			
Year of death	Norwegian Cause of Death registry		
Emigration	Norwegian Central Population registry		
Sibling	Norwegian Central Population registry		Parental information from the Norwegian Central Population Registry.

© 2026 Wolfova et al. *JAMA Neurology*.

eTable 2. Participants' characteristics according to hypertensive disorders of pregnancy, placental abruption and maternal diabetes

Variables	Preterm birth		Birthweight for gestational age[a]		
	No<br>N = 1 110 227	Yes<br>N = 57 987	SGA<br>N = 114 865	AGA<br>N = 939 324	LGA<br>N = 114 025
Participants' characteristics					
Calendar year of birth, No. (%)					
1967-1977	579 337 (52.2)	29 336 (50.6)	63 078 (52.0)	488 797 (49.8)	56 798 (52.0)
1978-1989	530 890 (47.8)	28 651 (49.4)	51 787 (48.0)	450 527 (50.2)	57 227 (48.0)
Sex, No. (%)					
Female	566,002 (51.0)	31 754 (54.8)	58 925 (51.1)	480 230 (51.4)	58 601 (51.1)
Male	544 225 (49.0)	26 233 (45.2)	55 940 (48.9)	459 094 (48.6)	55 424 (48.9)
Birth plurality, No. (%)					
Singleton	1 095 082 (98.6)	50 847 (87.7)	106 925 (98.5)	925 098 (99.9)	113 906 (98.5)
Multiple births	15 145 (1.4)	7 140 (12.3)	7 940 (1.5)	14 226 (0.1)	119 (1.5)
Congenital anomalies, No. (%)					
Yes	1 086 422 (97.9)	56 603 (97.6)	111 727 (97.9)	919 747 (97.8)	111 551 (97.9)
No	23 805 (2.1)	1 384 (2.4)	3 138 (2.1)	19 577 (2.2)	2 474 (2.1)
Maternal characteristics					
Country of origin, No. (%)					
Norway	1002,922 (90.3)	51 858 (89.4)	102 678 (90.3)	848 030 (91.3)	104 072 (90.3)
Other high income country	87 203 (7.9)	4 548 (7.8)	9 185 (7.9)	74 135 (7.4)	8 431 (7.9)
Middle- and low-income country	20 102 (1.8)	1 581 (2.7)	3 002 (1.8)	17 159 (1.3)	1 522 (1.8)
Age at the time of birth, Mean (SD)	26.2 (5.2)	26.2 (5.7)	25.5 (5.2)	26.1 (5.2)	27.5 (5.2)
Level of mother's education at the time of birth, No. (%)					
Primary	398 658 (36.5)	23 932 (42.3)	48 728 (36.5)	336 458 (33.2)	37 404 (36.5)
Secondary	524 355 (48.1)	25 004 (44.2)	50 648 (48.0)	443 257 (49.3)	55 454 (48.0)
Tertiary	167 786 (15.4)	7 590 (13.4)	12 820 (15.5)	142 879 (17.5)	19 677 (15.5)

Abbreviations. SGA = small for gestational age, AGA = appropriate for gestational age, LGA = large for gestational age, SD = standard deviation
[a] Calculated using distribution of the analytic sample

© 2026 Wolfova et al. *JAMA Neurology*.

eTable 3. Participants' characteristics according to hypertensive disorders of pregnancy, placental abruption and maternal diabetes

	HDP		Placental abruption		Maternal diabetes	
Variables	No<br>N = 1 118 616	Yes<br>N = 49 598	No<br>N = 1 163 436	Yes<br>N = 4 778	No<br>N = 1 165 552	Yes<br>N = 2 662
Participants' characteristics						
Calendar year of birth, No. (%)						
1967-1977	586 535 (52.4)	22 138 (44.6)	606 674 (52.1)	1 999 (41.8)	607 812 (52.1)	861 (32.3)
1978-1989	532 081 (47.6)	27 460 (55.4)	556 762 (47.9)	2 779 (58.2)	557 740 (47.9)	1 801 (67.7)
Sex, No. (%)						
Female	571 834 (51.1)	25 922 (52.3)	595 107 (51.2)	2 649 (55.4)	596 405 (51.2)	1 351 (50.8)
Male	546 782 (48.9)	23 676 (47.7)	568 329 (48.8)	2 129 (44.6)	569 147 (48.8)	1 311 (49.2)
Birth plurality, No. (%)						
Singleton	1 098 635 (98.2)	47 294 (95.4)	1 141 355 (98.1)	4 574 (95.7)	1 143 327 (98.1)	2 602 (97.7)
Multiple births	19 981 (1.8)	2 304 (4.6)	22 081 (1.9)	204 (4.3)	22 225 (1.9)	60 (2.3)
Congenital anomalies, No. (%)						
Yes	1 094 671 (97.9)	48 354 (97.5)	1 138 355 (97.8)	4 670 (97.7)	1 140 434 (97.8)	2 591 (97.3)
No	23 945 (2.1)	1 244 (2.5)	25 081 (2.2)	108 (2.3)	25 118 (2.2)	71 (2.7)
Maternal characteristics						
Country of origin, No. (%)						
Norway	1 009 428 (90.2)	45 352 (91.4)	1 050 479 (90.3)	4 301 (90.0)	1 052 466 (90.3)	2 314 (86.9)
Other high income country	88 237 (7.9)	3 514 (7.1)	91 363 (7.9)	388 (8.1)	91 526 (7.9)	225 (8.5)
Middle- and low-income country	20 951 (1.9)	732 (1.5)	21 594 (1.9)	89 (1.9)	21 560 (1.8)	123 (4.6)
Age at the time of birth, Mean (SD)	26.2 (5.2)	26.8 (5.7)	26.2 (5.2)	27.0 (5.5)	26.2 (5.2)	27.1 (5.4)
Level of mother's education at the time of birth, No. (%)						
Primary	404 876 (36.9)	17 714 (36.2)	420 625 (36.8)	1 965 (41.8)	421 684 (36.8)	906 (34.8)
Secondary	525 881 (47.9)	23 478 (48.0)	547 266 (47.9)	2 093 (44.5)	548 118 (47.9)	1 241 (47.7)
Tertiary	167 662 (15.3)	7 714 (15.8)	174 728 (15.3)	648 (13.8)	174 921 (15.3)	455 (17.5)

Abbrevations. HDP = hypertensive disorders of pregnancy, SD=standard deviation.

© 2026 Wolfova et al. *JAMA Neurology*.

eTable 4. Associations between maternal adverse pregnancy outcomes and the risk of multiple sclerosis in the offspring using alternative case definition (least 2 ICD code occurrences during the study period)

	Population analysis			
	**Incidence sample (N=1 166 731)**		**Prevalence sample (N=1 168 214)**	
Exposure	No. of events	Adjusted HR (95% CI)[a]	No. of events	Adjusted OR[b] (95% CI)
Preterm birth				
Yes	185	1.01 (0.87-1.16)	239	0.92 (0.80-1.05)
No	3822	1 [ref.]	5222	1 [ref.]
Birthweight for gestational age				
SGA	321	0.87 (0.78-0.97)	451	0.84 (0.76-0.93)
AGA	3228	1 [ref.]	4408	1 [ref.]
LGA	458	1.13 (1.03-1.25)	602	1.12 (1.03-1.22)
Placental abruption				
Yes	21	1.15 (0.74-1.81)	22	0.92 (0.79-1.06)
No	4371	1 [ref.]	5439	1 [ref.]
HDP				
Yes	161	0.88 (0.75-1.04)	203	0.92 (0.79-1.06)
No	4231	1 [ref.]	5258	1 [ref.]
Maternal diabetes				
Yes	19	2.11 (1.34-3.31)	21	1.93 (1.25-2.97)
No	4373	1 [ref.]	5440	1 [ref.]

Abbreviations. OR = odds ratio, HR = hazard ratio, CI = confidence interval, SGA = small for gestational age, AGA = appropriate for gestational age, LGA = large for gestational age, HDP = hypertensive disorders of pregnancy

Note. There were 4 011 cases with more than one MS ICD code between 2009 and 2019, and additionally 1 450 cases with more than one MS ICD within the first event registered in 2008.

© 2026 Wolfova et al. *JAMA Neurology*.

[a] Cox proportional hazard models were adjusted for sex, birth cohort (1967-1977 vs. 1978-1989), mother's age at the time of delivery, previous children, birth plurality, maternal education, and maternal country of origin.
[b] Logistic regression models were adjusted for calendar year of birth as a continuous variable, sex (female vs. male), mother's age at the time of delivery as a continuous variable, previous children (1, 2, 3, 4, 5+), birth plurality (singleton vs. multiple birth), maternal education (primary, secondary and tertiary), and maternal country of origin (Norway, other high income country, middle and low income country).

© 2026 Wolfova et al. *JAMA Neurology*.

eTable 5. E-values

	Population analysis					
	Prevalence sample (N=1 168 214)			Incidence sample (N=1 166 731)		
Exposure	No. of events	Adjusted OR[a] (95% CI)	E-value (point estimate; lower CI)[b]	No. of events	Adjusted HR (95% CI)[c]	E-value (point estimate; lower CI)[b]
Preterm birth						
Yes	254	0.92 (0.81-1.05)	1.40	198	0.99 (0.85-1.14)	1.20
No	5 546	1 [ref.]	-	4 114	1 [ref.]	-
Birthweight for gestational age						
SGA	502	0.87 (0.79-0.96)	1.50 (1.23)	369	0.88 (0.78-0.98)	1.54 (1.16)
AGA	4 679	1 [ref.]	-	3 469	1 [ref.]	-
LGA	619	1.09 (1.00-1.19)	1.40 (1.00)	474	1.13 (1.03-1.25)	1.51 (1.17)
Placental abruption						
Yes	23	0.98 (0.64-1.51)	1.13	21	1.17 (0.75-1.84)	1.63
No	5 777	1 [ref.]	-	4 291	1 [ref.]	-
HDP						
Yes	215	0.92 (0.80-1.05)	1.41	160	0.90 (0.76-1.05)	1.47
No	5 585	1 [ref.]	-	4 152	1 [ref.]	-
Maternal diabetes						
Yes	23	1.99 (1.32-3.01)	3.40 (1.97)	19	2.15 (1.37-3.37)	3.72 (2.08)
No	5 777	1 [ref.]	-	4 293	1 [ref.]	-

Abbreviations. OR = odds ratio, HR = hazard ratio, CI = confidence interval, SGA = small for gestational age, AGA = appropriate for gestational age, LGA = large for gestational age, HDP = hypertensive disorders of pregnancy

[a] Logistic regression models were adjusted for calendar year of birth as a continuous variable, sex (female vs. male), mother's age at the time of delivery as a continuous variable, previous children (1, 2, 3, 4, 5+), birth plurality (singleton vs. multiple birth), maternal education (primary, secondary and tertiary), and maternal country of origin (Norway, other high income country, middle and low income country).

[b] The limit of the confidence interval closest to the null is presented only for the exposures were OR or HR indicate a significant association.

© 2026 Wolfova et al. *JAMA Neurology*.

[c] Cox proportional hazard models were adjusted for sex, birth cohort (1967-1977 vs. 1978-1989), mother's age at the time of delivery, previous children, birth plurality, maternal education, and maternal country of origin.

© 2026 Wolfova et al. *JAMA Neurology*.

References

1. Engeland A, Bjørge T, Klungsøyr K, Skjærven R, Skurtveit S, Furu K. Preeclampsia in pregnancy and later use of antihypertensive drugs. *Eur J Epidemiol.* 2015;30(6):501-508.
2. Stene L, Eidem I, Vangen S, Joner G, Irgens L, Narve M. The validity of the diabetes mellitus diagnosis in the Medical Birth Registry of Norway. *Norsk Epidemiologi.* 2007;17.

© 2026 Wolfova et al. *JAMA Neurology*.
