# Supplementary material for: Maternal Pregnancy Outcomes and Offspring Risk of Adult-Onset Multiple Sclerosis
Source: JAMA Neurol. 2026 Jan 12;83(2):153–60. doi: 10.1001/jamaneurol.2025.5255 (PMC12797129; doi:10.1001/jamaneurol.2025.5255)
Supplement: Supplement 2. — Data sharing statement [file jamaneurol-e255255-s002.pdf]

Data Sharing Statement

Wolfova. Maternal Pregnancy Outcomes and Offspring Risk of Adult-Onset Multiple Sclerosis. *JAMA Neurol*. Published January 12, 2026. doi:10.1001/jamaneurol.2025.5255

Data
Data available: No

Additional Information
Explanation for why data not available: We are not providing data as this is already a secondary data study. Original data can be obtained from the original sources.
